# Health as a Secondary Property

**DOI:** 10.1093/bjps/axx014

**Published:** 2017-09-01

**Authors:** Alex Broadbent

**Affiliations:** African Centre for Epistemology and Philosophy of Science, Department of Philosophy, University of Johannesburg, Johannesburg, South Africa

## Abstract

In the literature on health, naturalism and normativism are typically characterized as espousing and rejecting, respectively, the view that health is objective and value-free. This article points out that there are two distinct dimensions of disagreement, regarding objectivity and value-ladenness, and thus arranges naturalism and normativism as diagonal opposites on a two-by-two matrix of possible positions. One of the remaining quadrants is occupied by value-dependent realism, holding that health facts are value-laden and objective. The remaining quadrant, which holds that they are non-objective but value-free, is unexplored. The article endorses a view in the latter quadrant, namely, the view that health is a secondary property. The article argues that a secondary property framework provides the resources to respond to the deepest objections to a broadly Boorsean account of natural function, and so preserves the spirit, though not the letter, of that account. Treating health as a secondary property permits a naturalistic explanation—specifically, an evolutionary explanation—of the health concept, in terms of the assistance such a concept might have provided to the survival and reproduction of those organisms that had it. (This approach is completely distinct from evolutionary and aetiological accounts of natural functions.) This provides the explanation, missing from Boorse's account, for the fact that function is determined with reference to the contribution to the goals of survival and reproduction, relative to the age of the sex of the species, rather than some other equally natural goals or reference classes.

1 Introduction
2 Two Ways to Disagree about Health
3 Secondary Properties
4 Health as a Secondary Property
5 Conclusion

## 1 Introduction

In the philosophical literature on health, there are two principal opposing positions: naturalism and normativism. These are typically set out as unitary and opposing positions in the following fashion:

Naturalists claim that health and disease are not determined by our *subjective* evaluations of a state, but are purely a matter of biological fact. Normativists reject this claim to *objectivity* and maintain that health and disease are essentially *value-laden*. (Kingma [2010], p. 242; my emphasis)

On this standard way of setting up the debate, naturalists hold that health is objective and value-free, while normativists hold that health depends upon humans by depending upon their values.

In light of this standard way of drawing the distinction, this article has two aims. The first is to highlight the fact that the naturalist and normativist are disagreeing along two dimensions. The naturalist is committed to two logically independent claims: that health facts are objective, and that they are value-free. The normativist is likewise committed to two logically independent claims: that health facts are non-objective (dependent upon health judgements or judgers in some inescapable way), and that they are value-laden. These commitments are logically distinct. This means that naturalists and normativists are not occupying positions at opposite poles of a debate, but rather are occupying two diagonally opposing positions in a two-by-two matrix. There are two other quadrants that are largely unexplored.

The second aim of this article is to investigate these under-explored boxes in the two-by-two matrix. These are the view that health is both objective and value-laden, and the view that it is neither objective nor value-laden. The former view corresponds to William Stempsey’s value-dependent realism. The other view, that health is neither objective nor value-laden, is to my knowledge completely unexplored. It can be made sense of by thinking of health as a secondary property. This is the view that I will explore and defend.

Section 2 distinguishes the two dimensions of the health naturalism debate—namely, objectivity and normativity—and shows how they are logically distinct. Section 3 offers a basic defence of the primary–secondary property distinction, and proposes that health is a secondary property that is neither objective nor value-laden. Section 4 offers a defence of a substantive account of health that could be used to flesh out the secondary property framework. It is simply the familiar Boorsean account of health as natural function, interpreted in the secondary property framework, which enables a rebuttal of the deepest objections to Christopher Boorse's substantive account.

Before continuing, let me limit the scope of this article in an important way. I am not considering views other than naturalism and normativism. There are views that seek to combine elements of naturalism and normativism (Wakefield [1992]), resulting in what we might call ‘weak’ or ‘mixed’ normativism, in contrast to ‘strong’ or ‘pure’ kinds that make health facts solely evaluative and that contain no independently specified empirical content (Boorse [1975], pp. 50–3); there are eliminativist views holding that we should rigorously distinguish state descriptions and normative claims, and eliminate talk about health unless clarified in this way (Ereshefsky [2009]); there are other views too (for helpful surveys, see Smart [2016], Chapters 1–2; Stegenga [forthcoming], Chapter 2).^1^ I am primarily focused on straightening the terms of the debate between naturalists and normativists, and on discovering new positions within that debate. I will not offer a detailed discussion of other views that seek to reject or alter the terms of the debate between naturalist and normativist.

Let me also forestall the possible objection that normativism has nothing at all to do with the objectivity or otherwise of health judgements. One might insist that the real meaning of ‘normativism’ is simply that health judgements are value-dependent, and that the objectivity or otherwise of value judgements is not part of the thesis. Boorse ([1975], p. 50) says that for the normativist, ‘all judgments of health include value judgments as part of their meaning’. I accept that in this early, partial definition of the term, Boorse does not mention the objectivity or otherwise of value judgements. However, the implicit definition of the term ‘normativism’, and of the opposing term ‘naturalism’, that arise from their actual usage in subsequent discussion clearly does include implications about the objectivity or otherwise of health judgements. This is evident from explicit remarks (as in the passage cited above, from Kingma) and from the nature of the points that are considered to support or pose difficulties for either of these positions, as I show in Section 2 below.

Let me also distinguish the central distinction drawn in this article from the four distinctions drawn by Elselijn Kingma ([2014], p. 590), who claims that ‘there is not one opposition between naturalism and normativism, but many’. She goes on to identify four domains within which one might be naturalist or normativist. These are distinctions between topics about which one might be a naturalist or a normativist. The binary opposition between naturalism and normativism remains; the correction Kingma proposes is to specify in which domain one is being a naturalist or a normativist, so as to avoid talking past one’s interlocutors. My contention is that naturalism and normativism conceal two distinct commitments, even within one domain.

## 2 Two Ways to Disagree about Health

Let us distinguish two dimensions of debate about health: the objectivity dimension and the normativity dimension. In this section, I explain what these dimensions are, and position the naturalist and normativist with respect to them.

The objectivity dimension of the debate concerns the extent to which health facts are judgement-independent. Naturalists believe that health facts are objective, or at least more objective than normativists take them to be. Naturalists hold that health facts do not depend on what we think about them, at least not in any important ways that are specific to the concept of health and distinguish that concept from scientific concepts such as mass, organism, or convection rolling. This is a clear and central part of Boorse’s ([1977]) motivation for putting health on a firm footing as a ‘theoretical concept’. Clearly, if the recognition of a fact is ‘a matter of natural science’, then the fact in question is, in some central and important sense, objective.

The normativity dimension of the debate concerns the extent to which health facts are value-laden. Naturalists deny that health facts are value-laden. Again, this is clear in Boorse’s ([1977], pp. 452–3) work:

On our view disease judgements are value-neutral, which is our second main result. If diseases are deviations from the species biological design, their recognition is a matter of natural science, not evaluative decision.

This passage indicates not only the naturalistic commitment to value-free health facts, but also the idea that there is a logical connection between health facts being independent, and being value-free.

This perceived connection is apparent in normativist work too:

By ‘disease’ we aim to pick out a variety of conditions that through being painful, disfiguring or disabling are of interest to us as people. No biological account of disease can be provided because this class of conditions is by its nature anthropocentric and corresponds to no natural class of conditions in the world. (Cooper [2002], p. 271)

Rachel Cooper holds an opposing position to Boorse’s on the objectivity dimension of disagreement: health facts depend on our interests. She is also committed to an opposing position to Boorse’s on the normativity dimension: she holds that the way health facts depend upon us is through bring painful, disfiguring, or disabling, which we single out because they are states we attach some (dis-)value to. This normative element is explicit in her definition of disease: ‘a condition that it is a bad thing to have, that is such that we consider the afflicted person to have been unlucky, and that can potentially be medically treated’ (Cooper [2002], p. 271). There is thus little doubt that there are self-describing naturalists and normativists who align themselves on the two dimensions I describe.

Discussions of the literature likewise tend not to discriminate between these two dimensions of debate. Kingma’s ([2014]) discussion of various domains of disagreement between naturalist and normativist begins by casting naturalism and normativism entirely as a disagreement along the normativity dimension. But the way that values are treated in the subsequent discussion, across each of the four domains she distinguishes, makes them non-objective. For example, there is discussion of whether states are ‘desired’ ([2014], p. 592); and there is a discussion of ‘wanted infertility’ to illustrate the difference between naturalism and normativism ([2014], pp. 593–4). Normative facts of this kind are clearly non-objective in an important sense—in contrast to the kind of normative fact that a moral realist might assert exists in the world, which both escape description in value-free language and are objective. Conversely, in the same discussion, the Boorsean naturalist is saddled—no doubt willingly—with the view that a health/disease line cannot ‘be so blurry that it could be drawn anywhere on the scale, because that would make the health/disease distinction random, and/or too prone to being determined by non-naturalist considerations and/or convention’ ([2014], p. 595). Thus although Kingma’s discussion starts with an apparently clean opposition between naturalism and normativism along the normativity dimension alone, the substance of the discussion clearly assumes that normativism and naturalism also differ along the objectivity dimension. The reference to ‘convention’, in particular, cannot be understood solely with reference to the normativity dimension. Among value theorists, it is by no means common ground that values are conventions; it is only among philosophers of medicine that this assumption appears to dominate.

Similar analyses apply to other discussions that promise to separate the two positions along one dimension alone. Dominic Murphy ([2015]) divides the debate along the objectivist versus constructivist line (thus selecting a dimension other than the one Kingma emphasizes), but characterizes constructivism with reference to a reliance not only on judgements, but on value judgements, which are again implied to be non-objective:

Although constructivists accept that disease categories refer to known or unknown biological processes they deny that these processes can be identified independently of human values […] Constructivist conceptions of disease are normative through and through.

Thus the broad consensus within much of the literature and among many commentators is that seeing health as (reasonably) objective goes hand-in-hand with seeing health as value-free, while seeing health as value-laden goes hand-in-hand with seeing health as ‘subjective’ (Kingma [2010], p. 242), or at least non-objective. Sometimes this connection is stated explicitly and seen as being logical in nature, as by Boorse and Cooper; sometimes it is not stated explicitly but is evident from the nature that normative facts are assumed to have, as by Kingma; and sometimes it is stated explicitly but with no logical connection identified, as by Murphy.

Accepting, then, that the objectivity and normativity dimension of disagreement are not usually separated, the obvious question is whether there are logical connections between the two dimensions, or whether they are logically independent. I now show that they are logically independent.

For clarity, let V stand for the claim that health judgements are value-laden, and O for the claim that they are objective. Then there are four possible logical connections to consider:

V⊢O

¬V⊢O

V⊢¬O

¬V⊢¬O

Let us take these in turn.

The claim that V⊢O (value-ladenness entails objectivity) would render the position of normativism logically incoherent, since normativism asserts that health judgements are value-laden and non-objective. I take it that normativism is not incoherent, or at least not for this reason. Moreover, there is no particular reason to suppose that this entailment holds. Therefore, we can conclude that it is not the case that value-ladenness implies objectivity.

The claim that ¬V⊢O (being value-free entails objectivity) is also implausible, on reflection, since there is surely more to objectivity than the mere absence of evaluative components.

The claim that V⊢¬O (being value-laden entails non-objectivity) is the most important of these possible entailments, since it connects value-ladenness and non-objectivity, the two ingredients of normativism; and, in the contrapositive, it connects being objective with being value-free, as naturalism supposes. We can see, however, that the entailment fails.

One might hold a species of moral realism according to which moral facts have a character that means they cannot be discovered by empirical inquiry, but, nevertheless, that they are objective facts. There are many kinds of moral realism (Railton [1986]; Shafer-Landau [2003]), and not all see moral facts as resistant to empirical discovery. However, at least one *prima facie* coherent view is that there are moral facts that are independent of what anyone thinks about them, and that these facts cannot be discovered by any scientific (or other empirical) inquiry, no matter how sophisticated or complete (Shafer-Landau [2003]; Parfit [2011]; Scanlon [2014]). Whether such a view implies either non-naturalism or anti-reductionism is a complex question (Railton [1986]), but since moral realism is not our topic here, let us assume that such a person is a non-naturalist about morality.

Suppose that a moral realist of this non-naturalist stripe agrees with the normativist that health facts depend on values. Such a person might be convinced that the notion of disease is so bound up in our ideas of what is good or bad for a person that it simply cannot be a matter of scientific fact alone. However, the moral realist will not (or need not) accept that this amounts to any sort of constructivism, nor any sort of eschewing of objectivity. She will disagree with Cooper that diseases are defined by whether we consider the afflicted person to be unlucky. She will (or, at least, may) regard disease facts as entirely objective. The fact that they cannot be discovered by natural science is not because they are not objective, but because they are non-natural. Just as science alone cannot tell us whether murder is wrong, science alone cannot tell us whether obesity is a disease. In both cases, the limitation arises from the fact that science cannot tell us about moral facts. According to this kind of (non-naturalist) moral realist, moral facts are out there nonetheless; they are non-natural facts to which science has no complete access.

William Stempsey defends a position of this kind, describing it as ‘value-dependent realism' (VDR). He summarises: ‘all facts in medicine, including facts about diagnosis, depend on values for their specification. However, […] there are objective values […] medical facts, even though they are built upon values, reflect an objective reality’ (Stempsey [2000], p. 34). VDR also appears to be the position of Kass ([1975], p. 23): ‘health, although certainly a good, is not therefore a good whose goodness exists merely by convention or human decree’.

The value-dependent realist agrees with the normativist on the normativity dimension but disagrees on the objectivity dimension. She thinks that health facts are both value-laden and objective. She thinks this without contradicting herself, because she puts the failure of science to discover the health/disease line down to the general failure of science to tell us about non-natural, but nonetheless perfectly real and objective, moral facts. The coherence of this view shows that there is no logical entailment from value-ladenness of health facts (which the VDRist accepts) to their non-objectivity (which the VDRist rejects). It also shows the failure of the equivalent entailment from the objectivity of health facts (which the VDRist accepts) to their being value-free (which the VDRist rejects).

There remains a further possible logical relation between value-ladenness and objectivity, namely, ¬V⊢¬O (being value-free entails being non-objective). This is implausible in its own right, and would also render naturalism incoherent, since naturalism holds that health judgements are both value-free and objective. I take it that naturalism is not incoherent, or at least not for this reason.

Thus there are no logical entailments between the two dimensions of disagreement at issue between naturalists and normativists, objectivity and value-ladenness (or normativity). I conclude, then, that they are logically independent.

In my view, it would aid clarity to move away from the accepted terminology, so that the component commitments are clearer in the positions formerly called ‘naturalism’ and ‘normativism’. Developing Stempsey’s terminology, we might rename naturalism ‘value-independent realism’ (VIR), and normativism ‘value-dependent anti-realism’ (VDAR). If we adopt this terminology, the availability of a fourth kind of position becomes obvious: value-independent anti-realism (VIAR). It helps to visualize the situation, by setting out a two-by-two matrix as is given below: 

**Figure:**
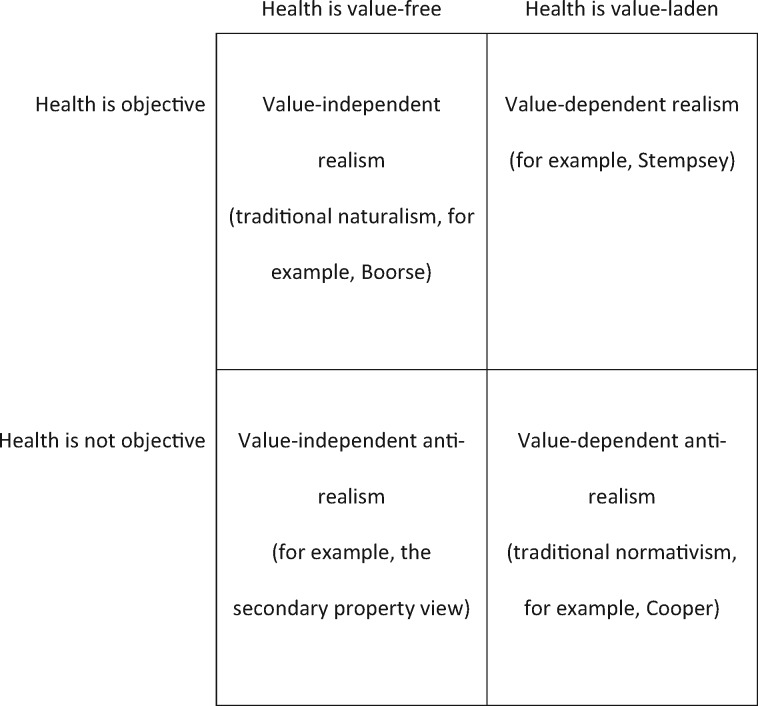


Having set out this framework, I intend to explore the quadrant that to my knowledge is entirely unexplored, namely, VIAR.

## 3 Secondary Properties

VIAR is the view that health facts are neither objective nor value-laden. What possible substantive position could give expression to these two commitments? There may be others, but the one that occurs to me is the view that health is a secondary property.

The primary–secondary property distinction is most famously associated with John Locke’s analysis of colour, although similar distinctions had been drawn before. Locke points out that certain properties, such as shape and mass, are independent of all observation, while others, such as colour, depend on observation in an essential way.^2,^3^^ The wavelength of a ray of light is a primary property, but the colour of a reflective surface is a secondary property. Many surfaces reflect electromagnetic radiation; we have visual apparatus that can detect some of that radiation in certain conditions and within that visible range, we cut the spectrum up in particular ways. To say that a surface is a certain colour is to say that, in certain conditions, typical humans would react to the radiation reflected from it in a certain way. The reaction does vary between individuals, but is not something we have control over, at either individual or social level. Some anthropologists have contended that there is some degree of convention in the way the colour spectrum is divided up. Nonetheless, the phenomenon of seeing colour in the first place is not naturally understood as conventional: it does not arise from social convention, but from the nature of our perceptual systems.

Thus, colours are both non-conventional and non-objective. There are clear non-conventional boundaries on colours. Maybe different societies draw the line between blue and green in different ways; maybe different individuals do so within a society. Personally I have the greatest difficulty telling whether others will call certain colours blue or green, even though I can see the difference between them. But irrespective of our cultural background or personal tendencies, we human observers do not see wavelengths in the infra-red or the ultra-violet range. At the same time, colour cannot be the objective property of being a wavelength of electromagnetic radiation. If colour were just wavelength of electromagnetic radiation, then it would exist wherever electromagnetic radiation exists; but there are no colours in the infra-red or ultra-violet parts of the spectrum.

Colour is something that arises in the course of an interaction between us human observers and the observer-independent world. Bat observers (at least the blind kind) would not acknowledge colours at all, just as we acknowledge no colours in the infra-red and ultra-violet spectra. Snake observers (which are deaf) would not have any distinctions between loud and soft sounds, harsh and sweet sounds, and so forth. Presumably, dolphin observers have sonar-related secondary properties, which we do not acknowledge. What secondary properties there are depends not just on the observed world, but on the nature of the observer in question.

In contrast, primary qualities are not dependent on observers. A red ball with a mass of 5 kg might feel heavier to one person than to another, but it will be 5 kg for both of them, and for anyone else, including persons in stronger or weaker gravitational fields. Indeed, it need not be 5 kg ‘for’ anybody at all in order to be 5 kg. It simply has a mass of 5 kg. However, the 5 kg red ball is, strictly speaking, red to humans. It is not red to bats, most of which cannot see, and it may not be red to bees, which can see parts of the spectrum that we cannot.

The fact that colour is a secondary property enables us to explain some otherwise puzzling things about it. One such thing concerns the fact that colours mix to form other colours. This is familiar, but it becomes strange when one learns that colours ‘are’ particular wavelengths of light, and moreover, that when two colours are mixed, the result is not a beam of light with a new wavelength, but two superimposed beams with two different wavelengths. Why, then, do we not see two colours at once? (Indeed, we cannot even conceive of this.) Why do we instead see one colour, corresponding to a wavelength of light that—we now learn—does not correspond to either of the wavelengths actually striking the eye, and is not in fact striking the eye at all?

Since we cannot conceive of seeing two colours at once, it helps to compare sound, which is also a wave (of a different kind, in a different medium) and which we also perceive. With sound, we are able to perceive two wavelengths at once. Suppose you play two notes on a piano at the same time, a C and the G above it. You do not hear an E, which is the average of the two wavelengths. You hear two notes, played together—in this case, a pleasantly bracing perfect fifth chord. Now consider what happens if you mix red and green light projected onto a white surface. You do not see a red and green ‘chord’—that is, you do not see two colours at once. You see one colour, yellow, corresponding (roughly) to the colour you would see if you projected a light with a wavelength equal to the average of the wavelengths of the red and the green light.^4^ Yet in the situation where you project red and green light, the reflected light hitting your eyes is not ‘averaged out’; the light hitting your eyes is of two wavelengths, corresponding to red and green, just as the sound hitting your ears when you play a C and a G is of two wavelengths.

Despite the many differences between light and sound radiation, the explanation for this phenomenon does not depend on those differences, but on the way we perceive colour. We have receptors in our retina called cones, which fire in proportion to the intensity of light of a certain wavelength striking them. These receptors are clustered around three different wavelengths, one each in wavelengths that we perceive as red, green, and blue. A red cone will fire most vigorously in red light, and less vigorously as the wavelength moves away from red; likewise for the other cones. In effect, and very crudely, our visual systems calculate the average of the intensity of the cones’ firing, and the colour we see arises from that average. This means that we cannot distinguish between a number of physically different situations. We cannot distinguish between a situation where a ‘yellow wavelength’ (to put it crudely, since a wavelength does not have a colour, as this explanation illustrates) strikes our eye, exciting red and green receptors to about the same extent and blue much less, and a situation where a ‘red’ and ‘green’ wavelength strike our eye simultaneously, which will also excite red and green receptors, and excite blue much less. This is why we see only one colour when colours are mixed. It is also why we can reproduce most of the visible spectrum by mixing just three colours (red, green, and blue). It explains colour-blindness: those who are red-green colour blind have only two kinds of cones, not the usual three. In short, the fact that colour is a secondary property explains a number of things that would be extremely difficult to explain otherwise.^5^

The primary–secondary property distinction was originally developed in connection with a distinction between perceptual properties (properties that have a feel, as it were) and non-perceptual properties. However, the essence of the notion of a secondary property is not dependent on sensory experience per se, but on the idea that a property might consist in part of our reactions to the world, as well as the nature of the world to which we react. My idea that health might be a secondary property was prompted by Peter Menzies and Huw Price’s ([1993]) suggestion that causation might be a secondary property. Causation is not a perceptual property: it is famously something that we cannot see, taste, and so forth. Menzies and Price are motivated by the thought that causation, like traditional perceptual secondary properties such as colour and feeling heavy, seems to us to be real, and yet is not apparent in the fundamental physics of the universe we inhabit. They suggest that this situation can be explained if causation is something that arises out of our interaction with the world as agents. They endorse an agency theory of causation, and use this to give shape to the suggestion that causation is a secondary property. In their view, causation depends on objective features of the world, but it also depends on us. Specifically, it depends on the fact that we are agents with the capacity to move around and intervene in worldly affairs. Along similar lines, Michael Dummett ([1978]) remarked that intelligent trees probably would not have developed the notion of causation.

If a view of this sort is correct, then the reason that causation is not discovered empirically, alongside mass, charge, spin, and the rest of the fundamental physical properties, is that it simply isn’t there. But if a view of this kind is right, then causation is certainly nothing like a social convention. It is something that depends both on us and the world; it depends on the place we occupy in the world, the kind of creatures we are, and on the nature of the world itself. Another world in which, by massive chance, thermodynamic asymmetries did not hold, need not violate the fundamental dynamical laws; and in such a world, a causal concept would probably have no grip (for excellent discussions in this domain, see Price [1996]; Price and Corry [2007]). Fires would dwindle into sparks just as often as sparks would light fires, matches would unlight and jump back into the box, and so on. We might still see sparks and fires as connected in such a world, but the asymmetry of causation, which is an essential aspect of the concept (Hausman [1998]), would be gone. Whatever it would be like to inhabit such a weird world, it seems highly plausible that the inhabitants—whatever they were like—would not share our concept of causation.

The point of this digression is not to establish that causation is a secondary property, but rather to set up a template for arguing that health is a secondary property. Health is unlike colour in that it is not a perceptual property. We do not ‘see’ health or its absence, at least not in the direct way that we see colour. In this way, health and causation are alike. They are alike, too, in that their objectivity is a topic of controversy. The template for arguing that a property like health or causation is a secondary property is: first, establish that it is hard to locate an objective basis for some property, which we nevertheless are strongly inclined to treat as objective; then offer an account of the property, according to which it arises from an interaction between us human observers and the world. This accounts for our inability to locate it objectively and, at the same time, for its apparent fixity, provided that the interaction between us and the world involves features that are suitably universal, or otherwise such as to be generally unnoticed.

The first part of the template is already fulfilled. It is clear that it is not easy to locate an objective basis for health facts. According to VDR and VIR (or, naturalism), there is such a basis, but even VIR-ists do not typically claim that it is an easy matter to say what it is; otherwise, we should not find it the subject of academic exertion. At the same time, everyone, including VDAR-ists (or, normativists), ought to admit that there is a strong inclination on our part to treat health as an objective property—as something that exists independently of our judgements. Maybe there are VDAR-ists who do not feel this strong inclination, but they must nonetheless accept, as a descriptive claim about fellow humans, that a large number of people talk and act as if health facts were objective. Thus, in health we have a property that we naïvely suppose has an objective basis, but whose objective basis is difficult to describe.

The second part of the template is to offer an account of health that shows how it is a secondary quality—on a par with Menzies and Price’s agency account of causation, or with the scientific account of colour vision in terms of cones. To this effort I now turn.

## 4 Health as a Secondary Property

According to Boorse’s well-known account, a body part or process is healthy when it contributes to the survival and reproduction of an organism—its ‘natural function’—to an extent that is at least statistically typical, relative to other organisms of the same age, sex, and species—its ‘reference class’ (Boorse [1977], [1997], [2011]). Very many objections that have been raised against Boorse’s account of health (comprehensively surveyed in Boorse [1997], [2014]). Those that I find most compelling are those alleging that Boorse lacks ‘an objective justification for his selection of reference classes’ (Kingma [2007], p. 131). Yielding on this point while insisting that disease is not value-laden puts us into the unexplored quadrant where the view that health is a secondary property lies. But let me first outline and endorse this objection that Boorse’s choice of reference classes is not objective.

The concern is that there is no proper justification for picking out the reference classes that Boorse selects. Kingma asks us to ‘imagine there are two candidate concepts for health. One is the BST [biostatistical theory, Boorse’s view], and one is XST. The XST is exactly like the BST, but has one more reference class: sexual orientation’ (Kingma [2007], p. 132). What empirical reason, Kingma asks, do we have for insisting on BST over XST? To extend the point, what about race? What about post code, make of car (if any), colour of hair, favourite colour? Is there any reason to pick out age and sex as health-determining, via the reference classes? Is there any natural reason?

Boorse’s ([2014], p. 695) reply is as follows:

As for choice of reference class, the one that I suggested medicine uses—an age group of a sex of a species—could hardly be a more biologically natural choice. Apart from one detail, the BST’s reference class is just one morphological type in the smallest taxon to which an organism belongs.

Boorse’s reply, then, is that his reference classes amount to a natural property, or perhaps to a property with the right degree of naturalness (Lewis [1983]); and it is this that justifies their choice. This reply does not answer the right question, however. The question is not whether reference classes are natural properties. Naturalness comes in degrees, if it comes at all; and as Boorse points out, a morphological type of the smallest taxon of a species appears to amount to a reasonably natural property. There are objectively significant similarities between property bearers; these can ground inductive inferences, may feature in high-level laws, and so forth. Perhaps they are not on a par with mass and charge for naturalness, but it would be unreasonable set the standard for a biologically natural property so high.

Putting it crudely, the question Kingma pushes is not whether the reference classes are objective, but whether the selection of these reference classes is objective. Thus the question is not whether an age group of a sex of a species is a natural property; the question is why this natural property should function as the reference class for our health judgements. It is hard to see how Boorse can do more than just assert that it should. He can support his position by appealing to the ability of his conception of health to capture actual medical usage, but even if he can defeat all counterexamples, there is an explanatory question that his theory does not answer.

Compare colour: The significance of certain wavelengths of light for a theory of colour perception does not arise merely from their being objective. The significance is explained by the fact that we are able to perceive and discriminate between some of them. When we understand this, we see that colour is not entirely an objective thing. Boorse’s stance is analogous to the stance of someone who seeks to defend an objectivist thesis about colour by repeatedly pointing out that the wavelength of red light is perfectly objective. The defence fails because it does not explain why we pick this objective property out in the way that we do when we apply the concept ‘red’. Once this explanation is supplied, we understand that colour is not wholly objective even though wavelengths are. Likewise, until we have an explanation for the connection of health with certain reference classes, health is not wholly objective even if the reference classes are. And even if we do have such an explanation, the explanation may—as in the case of colour—show us that health is not, in fact, objective, even if the reference classes are.

The secondary property framework offers a response to this line of objection. Indeed, there is no biological justification for these crucial claims. That is because health is a secondary property. It is a dispositional property of the natural functioning of organisms, roughly as defined by Boorse, to produce a certain response in us: the response we express in health judgements. To judge that something (body part or whole organism) is healthy is to judge that its contribution to the organism’s survival and reproduction is average or better, relative to the species.

The crucial question for Boorse, the one that I argue Boorse misses in his response to Kingma, is not whether this reference class is natural, or natural enough, but rather why this natural-enough reference class rather than one of many other possibilities. The secondary property framework enables us to offer an answer. There is nothing objectively special about an age group of a sex of a species. However, it is very special to us, because having average-or-better function for an age group of a sex of a species in relation to the goals of survival and reproduction is the same as having better chances in the grand evolutionary game. Assuming, as we must, that our concepts evolved along with the rest of us, it is not hard to imagine how thinking beings might have evolved a concept identifying the contribution that a body part is making to survival and reproduction. With such a concept, we would presumably be better able to devise and then take actions helping us survive and reproduce, along with the concept. This is the nature of an evolutionary advantage.

Relativization to an age group of a sex of a species is also advantageous, since it allows us to direct our actions appropriately depending on the prospects for an outcome. We do not worry that a baby cannot walk because this is normal for a baby, and in the normal course of things the baby will eventually walk. A concept that did not take this into account would have us wasting time on getting babies to walk too young. Likewise, it would have us lamenting the slow sprint of an aged person when there is nothing to be done about it, at least within the Palaeolithic context in which this concept evolved, and thus no point lamenting (although that person may lament it themselves).

Evolution-inspired defences of a form of VIR-ism (or, naturalism) have been attempted before. For example, Karen Neander ([2006]) and Benjamin Smart ([2016]) both seek to clarify the notion of natural function by reference to the function for which a body part or process evolved. Similarly, Mahesh Ananth’s ([2008]) book-length defence of an ‘evolutionary concept’ of health defends the idea that natural function can be explicated with reference to the goals of survival and reproduction. However, these approaches differ substantially from the sketch being advocated here. They attempt to explain natural function in terms of the contribution that a body part or process made, historically, to the survival and reproduction of organisms of the species. This is quite a different use of evolutionary explanation, since it aims to clarify the sense in which functions may be natural. My use, on the other hand, makes no claims on naturalness, but rather seeks to explain the fact we have a concept picking one equally natural set of functions out rather than another, in terms of the contribution that being able to pick them out would make to survival and reproduction.

I have no quarrel with an evolutionary account of natural function. The possible account that I have sketched is compatible with such accounts. But I do not think that, on their own, accounts such as those of Neander, Ananth, and Smart are an adequate response to the explanatory question posed by Kingma. They may show how the functions tracked by the health concept can be natural, but they do not explain why our health concept should track these natural functions. Showing that a function is natural is one thing; showing we have a reason to care about it is another. An evolutionary explanation of the health concept is one that shows how it helps with survival and reproduction; and a concept tracking these things would, one imagines, be relatively easy to explain, since it is relatively easy to imagine that it might confer an advantage in survival and reproduction.

I must stress that my evolutionary account of the concept of health is just a sketch. I have not made the detailed empirical argument that would be needed to fill the sketch out. My main purpose in this article is not to offer a substantive theory of health (supplying necessary and sufficient conditions for health), but an ontological theory (concerning the nature of health) (a similar distinction is often drawn between types of theory of causation, see Price and Corry [2007]). The evolutionary proposal just outlined is, thus, just an outline. A proper development would require a thorough evaluation of the kind of evolutionary advantage that a health concept might endow, and would need to guard against the risks of *ad hoc* explanation and the adaptationist fallacy that beset every evolutionary explanation of an actual trait, especially of social and cognitive ones.

Nonetheless, the sketch illustrates how a naturalistic account of health might be served by the secondary property approach. It is compatible with an evolutionary account of our coming to have the concept of health and is thoroughly naturalistic. Perhaps the deep problem with Boorse’s view is that the naturalism does not go deep enough: at some point, he just insists that certain goals relativized to certain reference classes are natural, without explaining how. If we accept that it is we, and not the world, that pick out the reference classes, then we can offer an explanation of why we do so. Contrary to the normativist, this explanation need have nothing to do with values: it may be entirely to do with the fact that creatures with this kind of concept survive and reproduce.

There are many more objections to naturalism (for summaries, consult Gifford [2011]; Smart [2016]) and I reiterate that I do not pretend to address them all. I do, however, believe that by distinguishing commitments to objectivity and to being value-free, it is possible to show how objections to the objectivity element of naturalism can be conceded without thereby necessarily conceding that health is value-laden. A secondary property view shows how this is possible, and an evolutionary account of the health concept would—if developed successfully—enable a value-free explanation of why that concept should track certain goals relative to certain reference classes. This would be thoroughly in keeping with the spirit of naturalism: one can concede that health facts are not objective without conceding that they are value-laden, and the kind of account that one can offer for the dependence of health facts on us may be thoroughly naturalistic.

## 5 Conclusion

Naturalism and normativism, as usually conceived, differ along two logically independent dimensions. This is often neglected in the debate between naturalists and normativists. A coherent position, VDR, can be constructed by combining naturalism's commitment to objective health facts with normativism's commitment to value-laden health facts. Another coherent position, VIAR, can be obtained by combining normativism's denial of objective health facts with naturalism's denial of value-laden health facts. I sought to explore the prospects for a view of this latter kind and, in doing so, endorsed a secondary property view of health.

I argued that Boorse’s substantive analysis of health as natural function could be defended from the difficulty of explaining the selection of reference classes within the secondary property framework, by pointing out that a concept that tracks the contribution of a trait to survival and reproduction is likely to confer an evolutionary advantage (that is, to aid survival and reproduction), and provided it is referenced to an age group of a sex of a species (since otherwise effort is wasted or misdirected on the elderly, the young, and so on). There is nothing special about contributing to the survival and reproduction relative to an age group of a sex of a species, as against various other equally natural potential reference classes. However, a concept picking out these rather than other reference classes might confer an evolutionary advantage. The secondary property framework allows us to admit that health is non-objective without conceding that it is evaluative. Indeed, it opens the door to an evolutionary explanation of the health concept that is thoroughly naturalistic in spirit, though this kind of evolutionary defence would require more development.

## Footnotes

^1^ Sometimes weak normativist views are called ‘hybrid’. For instance, Jerome Wakefield ([1992], p. 374) describes his own view of disease as harmful dysfunction in this way, because ‘harmful’ is analysed as a value-laden term, while ‘dysfunction’ is analysed as value-free. However, to describe the resulting composite as a hybrid view is misleading, in my view. There is no logical space between health facts being value-laden and value-free. It appears to me that this is one of the two core dimensions of disagreement between naturalist and normativist. The so-called hybridist is still a normativist (in this dimension) from the point of view of a naturalist, since she still holds that health facts are value-laden. Hence my preference for Boorse’s distinction between weak and strong forms of normativism (which, I note, may also helpfully admit of degrees). I am grateful to an anonymous referee for pressing this point.

^2^ He used the term ‘quality’ rather than property.

^3^ It may be that the distinction needs updating in the context of fundamental physics. In relativistic physics, mass varies with velocity, and velocity varies with frame of reference. In quantum physics, on standard interpretations, observer-dependence is common. I take it that the primary–secondary property distinction is still worth exploring as a conceptual distinction, with possible applicability at less fundamental levels, even if—like so much of our conceptual apparatus—it breaks down at the fundamental physical level.

^4^ I am talking about the mixing of lights projected onto a white surface—what is called ‘additive’ colour mixing. The kind that happens when you mix paints or other reflective substances is called ‘subtractive’ colour mixing. If you mix red and green paint, you do not get yellow but brown. This, again, is explained with reference to our visual systems.

^5^ Sound is different because we perceive it through the vibration of small hair-like structures inside our ears. These vibrate in the same way that the air vibrates, and thus if the air is vibrating with two different wavelengths at once, so do the hairs. Information about how the hairs are vibrating is neurally transmitted and is what ultimately becomes our perception of sound. With colour, however, nothing in the perceptual system directly corresponds to the wavelength of light; the information is inferred by the extent to which the light striking the eye approximates each of three different wavelengths.
